# Supplementary material for: asb5a/asb5b Double Knockout Affects Zebrafish Cardiac Contractile Function
Source: Int J Mol Sci. 2023 Nov 15;24(22):16364. doi: 10.3390/ijms242216364 (PMC10671462; doi:10.3390/ijms242216364)
Supplement: Supplementary file 1 [file ijms-24-16364-s001.zip › Supplementary Flie S1.pdf]

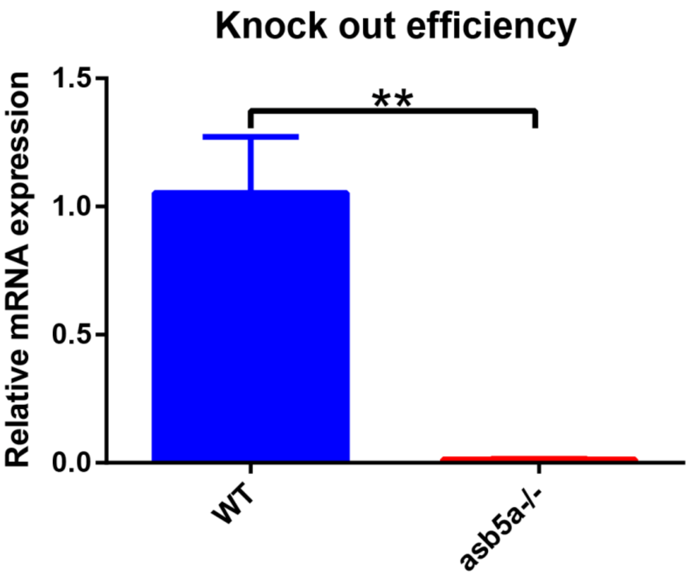

FigureS1. Validation of *asb5a* gene knockout efficiencyin zebrafish. The expression level of *asb5a*-mRNA in 48hpf homozygous embryos was identified by qPCR. Plotted data represent means ± standard error of the mean (SEM; n = 10, N=3).** $p < 0.01$.

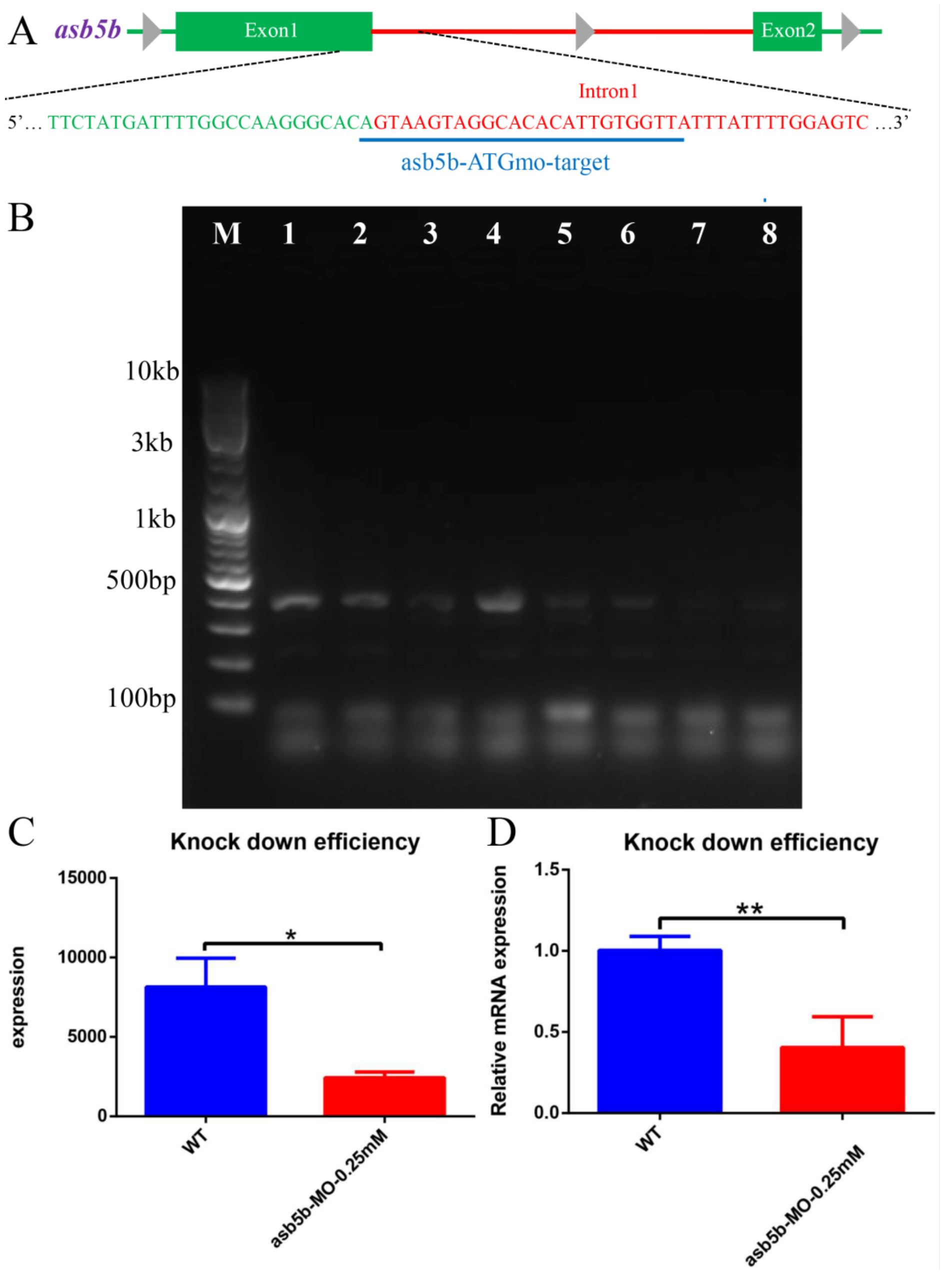

Figure S2. Identification of *asb5b* gene morpholino knockdown in zebrafish.**(A)**Target site design schematic of *asb5b*-ATGmo. Green represents the exons region, red represents the introns region, and blue represents the target site region.**(B)** The expression of *asb5b*-mRNA in 48hpf embryos was identified by RT-PCR. M: DNA marker bands；1-4: Four biological replicates of WT, 5-8: Four biological replicates of *asb5b*-ATGmo-0.25 mM were injected at the WT embryo one-cell stage.**(C)**Results of gray scale analysis electrophoresis. The results showed that at a low concentration of 0.25 mM, effectiveness knockdown of *asb5b*-mRNA expression levels compared with the WT group.**(D)**qPCR identified *asb5b*-mRNA expression levels in 48hpf WT embryos after injection of 0.25 mm low concentration *asb5b*-ATGmo.Plotted data represent means ± standard error of the mean (SEM; n = 10, N=4). * p< 0.05; ** p< 0.01.

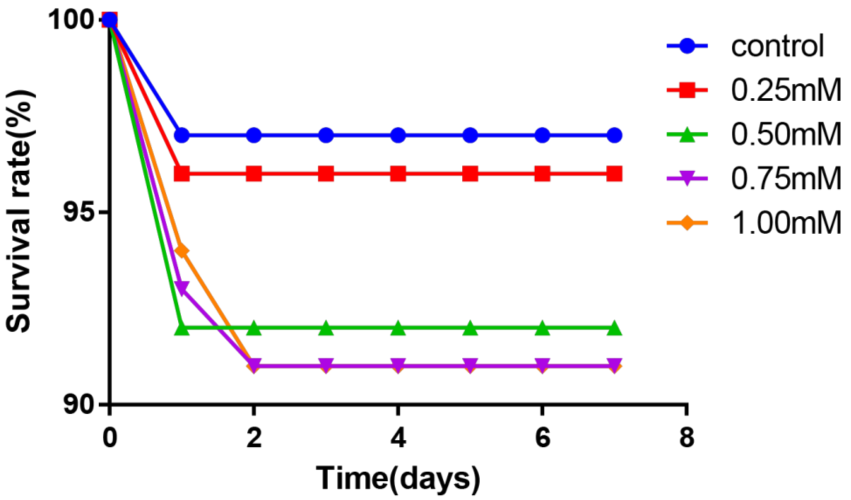

Figure S3. Survival curve of zebrafish with *asb5b*-ATGmo knockdown. Compared with WT Group, all the embryos with different injection concentration were broken membrane, normal body shape and heart development after 3dpf. Overall survival rates were above 90%, as shown above.

Table S1: Primers used for gene knockout and their sequences.

Primer	Sequences (5′→3′)
sgRNA-F1	tgTAATACGACTCACTATAGGgaatggcctgtactcctctgGTTTTAGAGCTAGAAATAGC
sgRNA-F2	tgTAATACGACTCACTATAGGagttctatgacaatggtcaaGTTTTAGAGCTAGAAATAGC
sgRNA-R	aaGCACCGACTCGGTGCCACT
asb5a -KO-F	ACTAGACATTTTGTATATAAACTTAT
asb5a -KO-R	GTGCCCTTTAAATCTATTTTTG

Note: tg was the protective base, and the sequence of T7 promoter was TAATACGACTCACTATA. GG was added to improve the transcription efficiency of T7 promoter. The horizontal part of the base is the target site sequence,GTTTTAGAGCTAGAAATAGC is the upstream of the sgRNA backbone sequence, and aais the protective base.

Table S2: *asb5b*-ATGmo concentration gradient design.

asb5b-ATGmo Original fluid	Nuclease-Free Water	Final concentration
1μL	3μL	0.25milliMolar(About 1.2 ng/embryo)
1μL	1μL	0.50milliMolar(About 2.5 ng/embryo)
3μL	1μL	0.75milliMolar(About 3.6 ng/embryo)
1μL	0μL	1.00milliMolar(About 4.9 ng/embryo)

Note: *asb5b*-MO powder 300 nanomoles (about 2.5 mg) . ADD 0.3 ml Nuclease-Free

Water (about 8.33 ug/ul) to the powder. About 1,700 embryos can be injected per 1 ul of solution.

Table S3: Primers used for WISH and their sequences.

Primer	Sequences (5′→3′)
asb5a–WISH-F	agttcctatgtgcccgcaagctg
asb5a–WISH-T7R	CCCTAATACGACTCACTATAGGGttatctgtattgtagaaagttcttc
asb5b–WISH-F	ggcctttcggctctaatacca
asb5b–WISH-T7R	CCCTAATACGACTCACTATAGGGttatctgtattgaaggaagc

Table S4: Primers used for qPCR and their sequences.

Primer	Sequences (5’→3’)
actb1–qPCR-F	TCCAGCCTTCCTTCCTGGGTAT
actb1–qPCR- R	GTGGAAGGAGCAAGAGAGGTG
cox6a2–qPCR-F	ACTGTCCTTCGTGTTAGCCC
cox6a2–qPCR-R	AGTGGGCAGAGCGTTTGTAT
cox7c–qPCR-F	ACAAGCTGTTCGTCGGTTTG
cox7c–qPCR-R	ATGAAGGGGAAGGCGAATCC
tnnc1a–qPCR-F	GGATGTTAGGCCAGAACCCT
tnnc1a–qPCR-R	TGGCTTCACCTGTAGCTTCC
tnnc1b–qPCR-F	CAGCGGTGGAGAACTTGACA
tnnc1b–qPCR-R	CGCACCATCATGACCAAGAAC
tnnt2a–qPCR-F	GATCGACGCAAACCTCTGGA
tnnt2a–qPCR-R	GGTCACTGACTCTGTTCCTCA
acta1a–qPCR-F	ATGCCATCATGCGTTTGGAC
acta1a–qPCR-R	GGAAGCTCGTAGCTCTTTTCG
actc1a–qPCR-F	GGAAATGAGCGTTTCCGCTG

actc1a–qPCR-R	GGCCAGAGCAGTGATTTCCT
actc1b–qPCR-F	CAATGGCTCTGGCTTGGTCA
actc1b–qPCR-R	ACTTCAGGGTCAAGATACCTCTC
actc1c–qPCR-F	TATGTGGGAGACGAGGCTCA
actc1c–qPCR-R	CGGGAACGTTGAAGGTCTCA
cacng1a–qPCR-F	TGAGTCCTCGAGTGGAAACAAT
cacng1a–qPCR-R	GAAGAGGGCTATGGCAGCAC
cacng2a–qPCR-F	ATGGCTGAAATGGTGGGTGT
cacng2a–qPCR-R	GAAGGTAGAGCCCCGAAACC
cacna1fb–qPCR-F	ACCCTACTGCTCTACAGGCG
cacna1fb–qPCR-R	GGCTTCTGCTTAAGGGCTCA
cacna2d1a–qPCR-F	CCCATCGATTGGAGCCATCA
cacna2d1a–qPCR-R	GGACCCAGGTTCTTAGGCTT
cacna2d2b–qPCR-F	ACACCACAGTACAAGCTCGG
cacna2d2b–qPCR-R	ATGTAGCGACTTGCCTCGTC
cacna2d3–qPCR-F	GGGCAAACTGGTGTCAATGG
cacna2d3–qPCR-R	GTGAGCTGCGGAGAAAAGATT
cacna2d4a–qPCR-F	CAATTGGCCAGATCGCAAGG
cacna2d4a–qPCR-R	GCACACTGTCCATGTAAGCC
cacna2d4b–qPCR-F	GGCCTGACAAGAAGGTACGA
cacna2d4b–qPCR-R	CTTGAACACCACACTGTCCATA
cacnb1–qPCR-F	GTCCTCCTGAGCTGTTTGACA
cacnb1–qPCR-R	GAAGTGAGCACCTGTACCTGC
cacnb2a–qPCR-F	AACAACATTGGCGGTTCAGC
cacnb2a–qPCR-R	ATGAGATTCCCGTTCCAGGC
tpma–qPCR-F	AGTACTCAGCTAAGGAGGACAAAT
tpma–qPCR-R	CTCGCTGATGGCTTTGTACTTG
cox7b–qPCR-F	AGTCTCGACCGCTCATTTCC
cox7b–qPCR-R	CTTCTGTCGAACAGCCACCT

myh7–qPCR-F	GGAGCAGCAGCTTCTTACCT
myh7–qPCR-R	CTGGGGGTGAATGTCAGCTT
cox5b–qPCR-F	GAGCTGTGGTTTTGCGTCAC
cox5b–qPCR-R	TGAGGGTCTTGTTTGGAGCC
cox8b–qPCR-F	ATGTCCGGCTTCAATCGCT
cox8b–qPCR-R	GGATGGTCACAAACATGACCG
uqcrc1–qPCR-F	CATCACATTCGGGGGTGGAA
uqcrc1–qPCR-R	TCGGTCACTGTGGTGCATAC
uqcrfs1–qPCR-F	GGGTCTACCCTTGTGGTTGG
uqcrfs1–qPCR-R	TTCTCTGTTCTGTGGCGGAC
myl4–qPCR-F	ACAGAACGCCAGCAAGTGAA
myl4–qPCR-R	TGTGCCCTGATCTTTGGACC
uqcr10–qPCR-F	ATGGCGCTCTCTAAAACGGT
uqcr10–qPCR-R	GTTTCCAGAGTTTCCCCCGA
cox7a2l–qPCR-F	AAGGGCAAATGTACCCTCCG
cox7a2l–qPCR-R	CTGTCTGGAACACCTCGCTT
cox6b2–qPCR-F	GATGCCCGTTTCCCAAACAC
cox6b2–qPCR-R	CCCACCCAGCTAATTGGACA
asb5a–qPCR-F	TCATGAGGCCTGCCAATGTAA
asb5a–qPCR-R	TGCCCCAAATTCCAGCAAGA
asb5b–qPCR-F	CGTGGTTAAAGGAAATCGTAAGG
asb5b–qPCR-R	TTTGCACTGTAACCCTGTGA

Note: Red highlights and yellow highlights are *asb5a*$^{-/-}$:*asb5b*-ATGmo double knockout group compared with the WT group analyzed the enriched top 20hub genes using STRING database and Cytoscape software; Red highlights and green highlights are *asb5a*$^{-/-}$:*asb5b*-ATGmo double knockout group compared with the *asb5a*$^{-/-}$group analyzed the enriched top 20hub genes using STRING database and Cytoscape software.
